# Postoperative dystocia of the gastric bursa after LRYGB: A case report

**DOI:** 10.1097/MD.0000000000035077

**Published:** 2023-10-27

**Authors:** Hang Yu, Xing Kang, Xitai Sun

**Affiliations:** a Department of General Surgery, Nanjing Drum Tower Hospital Clinical College of Nanjing University of Chinese Medicine, Nanjing, China; b Department of General Surgery, Drum Tower Hospital Affiliated to Nanjing University Medical School, Nanjing, China.

**Keywords:** laparoscopic Roux-en-Y gastric diversion, post-operative re-fattening, revision surgery

## Abstract

**Rationale:**

Laparoscopic Roux-en-Y gastric diversion is one of the most widely used surgical procedures for weight reduction and metabolic surgery, which is a hybrid approach to restrict intake and reduce absorption. Despite the successful completion of laparoscopic Roux-en-Y gastric diversion, 10% to 20% of patients still experience regained body mass or other complications.

**Patient concerns:**

The patient had regained weight after all the RYGB surgeries, and after diet and exercise control, the results were not good, so she came to our department for treatment.

**Diagnoses:**

Dilatation of the gastric pouch was observed on iodinated water imaging of the upper gastrointestinal tract and on abdominal CT.

**Interventions:**

We report 2 patients with dilated gastric bursa after RYGB, both female, who underwent gastric diversion revision.

**Outcomes:**

Both patients in this case underwent laparoscopic gastric diversion correction to improve weight rebound. Their quality of life improved significantly after treatment. There were no grade 3/4 treatment-related adverse events during the treatment period.

**Lessons:**

The above cases suggest that patients who regain weight after RYGB should routinely undergo preoperative upper gastrointestinal endoscopy and upper gastrointestinal iodine hydrography in order to observe the muscle tone of the patient’s gastric bursa and the degree of dilatation of the gastrointestinal anastomosis and consider whether to correct the dilated gastric bursa intraoperatively before converting to LSG.

## 1. Introduction

Laparoscopic Roux-en-Y gastric diversion (LRYGB) as one of the widely used clinical procedures for weight loss metabolic surgery is a hybrid weight loss metabolic surgery modality that restricts intake combined with reduction of absorption.^[1]^ Despite successful completion of LRYGB, 10% to 20% of patients still experience weight regain or other complications including recalcitrant malignancy, severe dumping syndrome, chronic pain, weight loss failure and recurrent anastomotic ulcers.^[2]^ Gastrointestinal anastomotic dilatation is one of the more common complications after LRYGB, and herein, we report a rare phenomenon of gastric bursa dystrophy through 2 cases of post-gastric diversion gastrointestinal anastomotic dilatation and propose a new treatment experience for this phenomenon.

## 2. Materials and Methods

**Figure 1.**
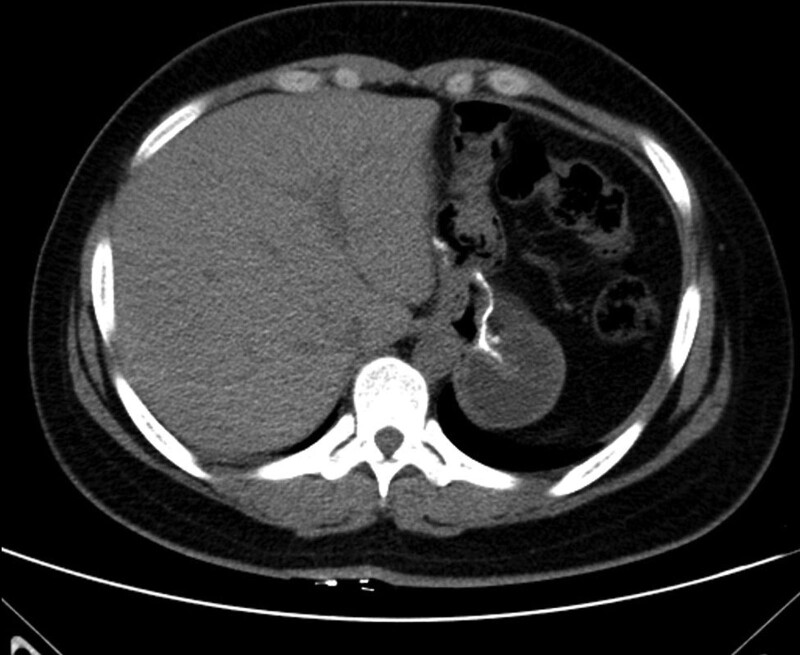
Preoperative computed tomography (CT) showed dilated gastric bursa.

Case 1: 31-year-old female, BMI: 37.6 kg/m^2^, obesity, nonalcoholic fatty liver disease, underwent LRYGB in our hospital in February 2017. All preoperative tests were normal at upper gastrointestinal endoscopy, and intraoperatively the plasma membrane was opened between the first and second branches of the gastric branch of the left gastric artery close to the gastric wall, preserving about 30 mL of the proximal gastric sac with a 100 cm gallbladder-pancreatic limb and 250 to 350 cm of GI limb. The gastrojejunal anastomosis is 1 to 2 cm wide in diameter. The gastrojejunostomy is then closed in 2 layers by a hand-sewn technique. The same technique was used to construct the jejuno-jejunostomy. He was discharged 3 days after surgery on a clear liquid diet. More than 2 years after surgery, the patient complained of progressive weight gain and was readmitted to the hospital. A complete laboratory examination, computed tomography scan of the chest and abdomen was performed. Laboratory tests were normal, while computed tomography revealed significant dilatation at the gastrointestinal anastomosis (Fig. 1). We considered that the patient had hypertrophy due to dilatation of the gastrointestinal anastomosis and loss of tone in the gastric bursa. Therefore, we decided to perform a laparoscopic gastric diversion revision of the patient to correct the LRYGB to Laparoscopic Sleeve Gastrectomy (LSG).

**Figure 2.**
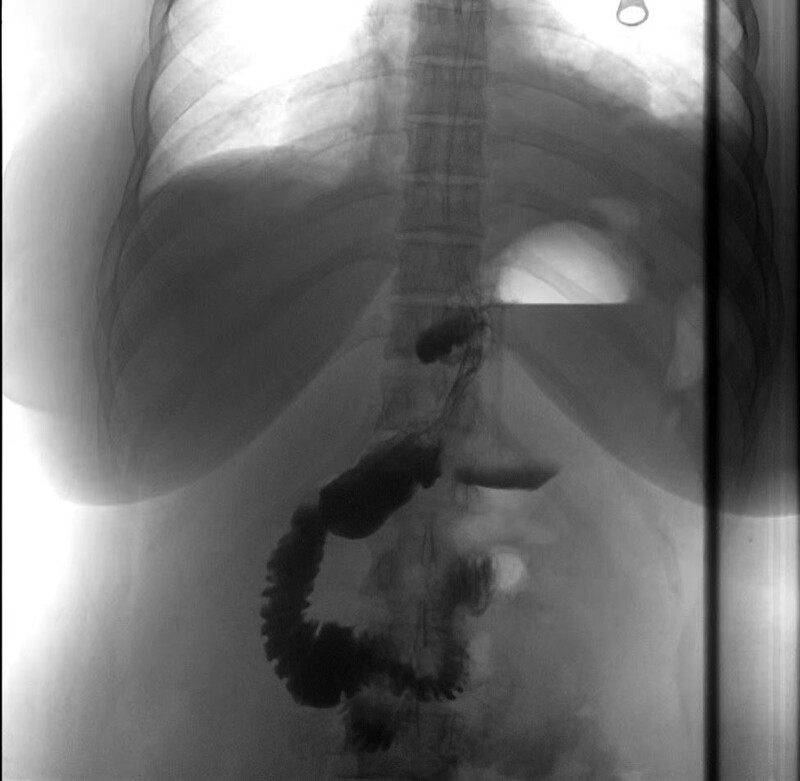
Iodine hydrography of the upper gastrointestinal tract.

Case 2: 36-year-old female, BMI: BMI: 36.0 kg/m^2^, obesity, nonalcoholic fatty liver disease, underwent LRYGB at our hospital in September 2016. All preoperative examinations were normal on upper gastrointestinal endoscopy, and the procedure was performed as above. Three years after surgery, the patient came back to our hospital due to progressive weight gain, and the relevant investigations were completed, and the upper gastrointestinal angiography showed dilated gastric bursa (Fig. 2). The clinical diagnosis considered dilated gastrointestinal anastomosis and dystocia of the gastric bursa. Laparoscopic correction of gastric diversion was performed in November 2019 to correct LRYGB to LSG.

## 3. Results

**Figure 3.**
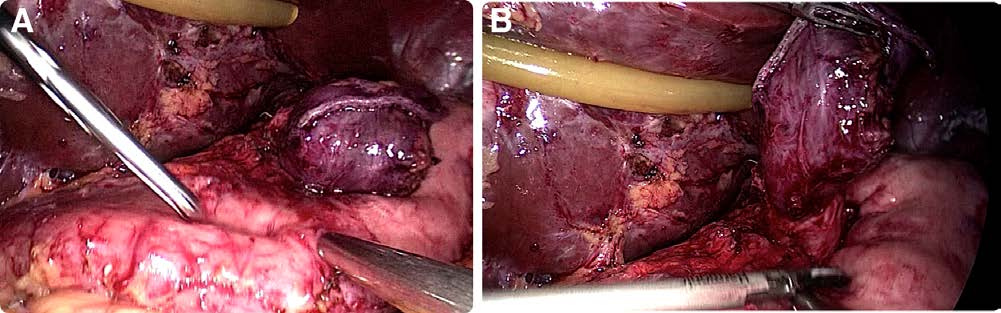
Gastric bursa dilatation.

**Figure 4.**
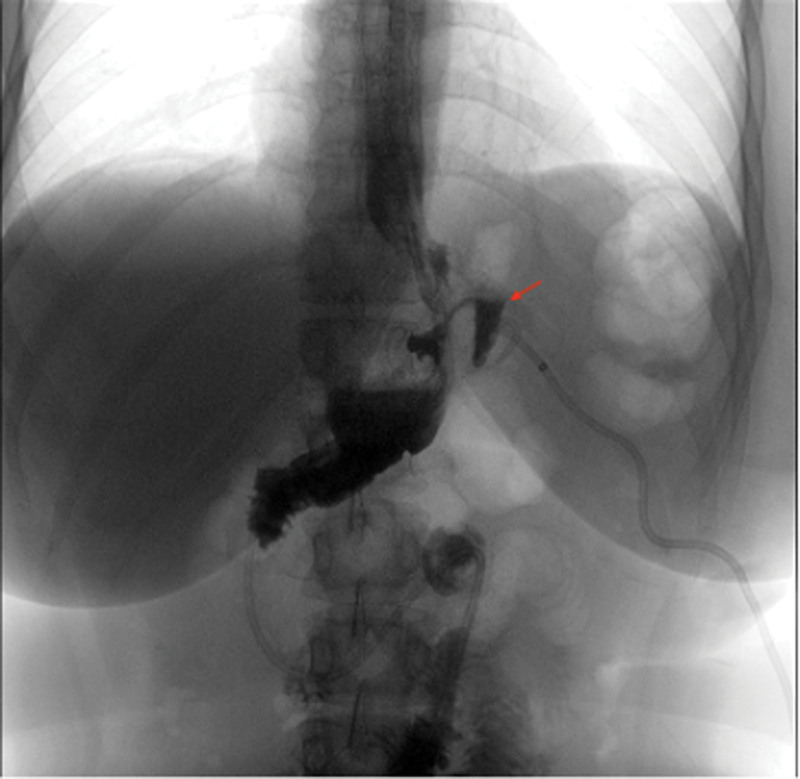
Postoperative upper gastrointestinal tract angiography.

During the laparoscopic exploratory examination, we observed the dilated gastrointestinal anastomosis as well as the gastric bursa (Fig. 3). The adhesions in the surgical area were separated, the liver suspension band was placed into the abdominal cavity, the left outer lobe of the liver was suspended to reveal the surgical area, the original gastrointestinal anastomosis and the open stomach were freed, and the intestinal collaterals were closed at 4 cm distal to the gastrointestinal anastomosis with a cutting closure device. The original gastrointestinal anastomosis was completely excised, the original open stomach was freed, the adhesions were separated, the greater omentum was lifted, the greater omentum was incised in the middle of the greater curvature of the stomach against the gastric wall (within the vascular arch of the omentum) with an ultrasonic knife, the distal end was freed to 4 cm proximal to the pylorus, the distal end was freed from the fundus, the short gastric vessels were clamped, the posterior gastric adhesions and vessels were fully freed, the gastric wall was incised on the side of the lesser curvature near the broken end of the residual stomach, and a 36F guide tube was inserted through the mouth during the operation and passed through the original gastric The 36F guide tube was inserted through the mouth and passed through the original gastric bursa to the pylorus, and under the guidance of the guide tube, a Johnson cutter was placed to perform sleeve-like gastric formation from 4 cm above the pylorus to the fundus of the stomach, and 405 Johnson barbed sutures were used to perform end-to-end anastomosis between the proximal gastric bursa and the distal sleeve-shaped remnant stomach, and the pulpy muscle layer was reinforced with sutures to restore gastric coherence. On the first postoperative day, the patient was given a small amount of water, which was tolerated by the patient. On the second day after surgery, the patient abdominal drainage tube was removed. On the third postoperative day, iodine hydrography of the upper gastrointestinal tract was performed to observe the patency of the gastrointestinal tract, and in both patients, iodine hydrography of the upper gastrointestinal tract showed patency of the gastrointestinal tract and no gastric fistula. Notably, the contrast findings revealed a flow of contrast to the dilated gastric bursa (Fig. 4).

## 4. Discussion

To our knowledge, a published case of LRYGB with dilated gastrointestinal anastomosis and dystonic gastric bursa corrected to LSG is the first one. 1 to 1.5 years after LRYGB, the patient gastrointestinal anastomosis will be dilated to 2 to 3 cm, and poor postoperative dietary control in obese patients and excessive eating will cause dilatation of the gastric bursa and gastrointestinal anastomosis and subsequent regain of weight. Both patients in this case had their LRYGB corrected to LSG by laparoscopic correction of gastric diversion to improve regained weight. However, we found some problems from these 2 cases. In the postoperative iodine hydrography of the upper gastrointestinal tract, we found dilated gastric bursa, which we defined as the phenomenon of dystocia of the gastric bursa. It is different from the common clinical gastric bradycardia, which cannot be treated by pro-gastrointestinal dynamics drugs. Furthermore, in this case, we believe that preoperative upper gastrointestinal endoscopy as well as upper gastrointestinal iodine hydrography should be routinely performed on the patient to observe the dystonia of the gastric bursa and the degree of dilatation of the gastrointestinal anastomosis in order to consider whether to correct the dilated gastric bursa intraoperatively before converting it to Laparoscopic Sleeve Gastrectomy.

Dystocia of the gastric bursa after LRYGB is uncommon. The occurrence of this phenomenon often leads to a lack of significant postoperative weight loss and a tendency to regain weight. Therefore, it is more important to focus on the dilatation of the gastric bursa when performing revision surgery for this reason. In addition, the American Society for Metabolic and Bariatric Surgery classifies 3 types of revision surgery: revision surgery, repair surgery, and recovery surgery.^[2]^ For LRYGB, commonly used revision procedures include gastric banding, in which a gastric band is placed over the gastrojejunal anastomosis to increase restriction of intake, and increased malabsorption, which converts LRGB to Biliopancreatic shunt-duodenal diversion. Gastric banding has the advantage of being simple to perform and is mainly used to treat dumping syndrome that occurs after LRYGB, and is less effective in correcting inadequate weight loss than conversion to BPD is not as effective as conversion to BPD. In contrast, although BPD can improve the efficacy of weight loss, it has the disadvantage of being operationally complex, with a certain rate of reoperation failure and high surgical risk. Therefore, for the above 2 patients, we chose to restore the LRYGB to its normal anatomy, that is, modified to LSG. A retrospective study by Moon et al similarly concluded that for inadequate weight loss or severe complications after LRYGB surgery, it is reasonable to restore the LRYGB to its normal anatomy.^[3]^

With the rapid development of bariatric metabolic surgery in recent years, the number of bariatric surgery cases has been increasing, and the number of some patients who have poor postoperative results, regain weight and complications and need to undergo revision surgery has also been increasing. Therefore, preoperative evaluation and individualization of the surgical plan is a crucial step. Common preoperative evaluations include detailed history taking and physical examination, blood tests, upper gastrointestinal examinations, abdominal ultrasound and *H pylori* testing.^[4]^ In addition, postoperative management and follow-up after revision surgery should also receive attention. A randomized controlled trial of an 18-month artificial intervention to improve the lifestyle of patients after bariatric surgery found that this intervention could bring potential psychosocial and behavioral benefits, largely improve patients’ postoperative compliance, further improve physical performance in most patients, and also help to analyze and study the factors influencing poor outcomes after bariatric surgery.^[5]^

## 5. Conclusion

The conversion from RYGB to SG successfully resolved the phenomenon of dilated gastrointestinal anastomosis after RYGB, and better controlled the patient postoperative weight condition. In contrast, this method needs to be carefully selected for patients with severe chronic complications after RYGB. In addition, through the above 2 patients, we observed a rare phenomenon of gastric bursa dystrophy and proposed a new treatment idea for the subsequent occurrence of this phenomenon.

## Author contributions

**Writing – original draft:** Hang Yu.

**Writing – review & editing:** Xing Kang, Xitai Sun.
